# On the Potential Role of the Antioxidant Couple Vitamin E/Selenium Taken by the Oral Route in Skin and Hair Health

**DOI:** 10.3390/antiox11112270

**Published:** 2022-11-17

**Authors:** Joël Pincemail, Smail Meziane

**Affiliations:** 1 CHU of Liège, Platform Antioxidant Nutrition and Health, Pathology Tower, 4130, Sart Tilman, 4000 Liège, Belgium; 2 Institut Européen des Antioxydants, 54000 Nancy, France

**Keywords:** skin aging, skin disorders, hair, selenium, vitamin E

## Abstract

The relationship between oxidative stress and skin aging/disorders is well established. Many topical and oral antioxidants (vitamins C and E, carotenoids, polyphenols) have been proposed to protect the skin against the deleterious effect induced by increased reactive oxygen species production, particularly in the context of sun exposure. In this review, we focused on the combination of vitamin E and selenium taken in supplements since both molecules act in synergy either by non-enzymatic and enzymatic pathways to eliminate skin lipids peroxides, which are strongly implicated in skin and hair disorders.

## 1. Introduction

Aging is a complex process involving progressive physiological changes in an organism that leads to senescence. This results in the progressive decline in the resistance to stress and other cellular damages, causing a gradual loss in cellular functions and resulting eventually in cell death. López-Otín et al. [1] identified nine cellular and molecular hallmarks of aging being divided into three groups. The primary hallmarks include genomic instability, telomere attrition, epigenetic alterations, and loss of proteostasis. Antagonistic hallmarks include deregulated nutrient sensing, cellular senescence, and mitochondrial dysfunction. Finally, integrative hallmarks include stem cell exhaustion and altered intercellular communication.

As early as the1950s, Harman [2] proposed that increased production of free radicals by mitochondria was a driving force in the aging process (Mitochondrial Free Radical Theory of Aging or MFRTA). Following modifications of this theory, Sohal and Orr [3] and Viña et al. [4] proposed the cell signaling disruption theory of aging. In the case of excessive production of reactive oxygen species (ROS), aging only occurs if there is a disruption in the whole signaling network dependent on physiological ROS for receiving and transmitting signals in order to process information to cells [5].

With the largest surface area on the body, the skin is extremely sensitive to both intrinsic and extrinsic aging [6]. Intrinsic aging is influenced by physiological factors, it is specific to each individual and it is genetically programmed. Extrinsic aging is influenced by external factors and can lead to premature aging of the skin. Both processes are associated with an increased production in ROS, thus leading to oxidative stress (OS) [7,8]. Similar to the skin, the scalp is also subject to ageing, which manifests as a decrease in melanocyte function or graying, and a decrease in hair production or alopecia. As reported by Trüeb [9], increased oxidative stress plays a pivotal role in the gradual loss of pigmentation.

In this context, low molecular weight antioxidants and trace elements that are able to interfere with ROS obtained from diet [10,11,12] or supplements [13,14,15,16,17] are believed to represent a good strategy to prevent hair aging and to delay skin damage, such as fine lines and wrinkles, dullness, uneven skin tone, dry skin, age spots, rough skin texture, visible pores and, potentially, and skin cancer.

Among all the antioxidants, vitamin E/selenium have a single profile. Indeed, both molecules have the particularity to act in synergy in eliminating lipid peroxides (ROOH) [18] known to be associated in the development of skin and hair diseases [9,19]. Vitamin E can directly interact with lipid free radical (ROO^•^) [20], while selenium is the main co-factor of glutathione peroxidase (GPx), a key enzyme that reduces lipid peroxides into harmless molecules [21]. The aim of the present review was to examine how the combination of selenium and vitamin E taken orally could potentially contribute to skin protection. Before that, it is important to inform dermatologists about the link between oxidative stress, antioxidants and skin aging and diseases.

## 2. Oxidative Stress Definitions

**Figure 1:**
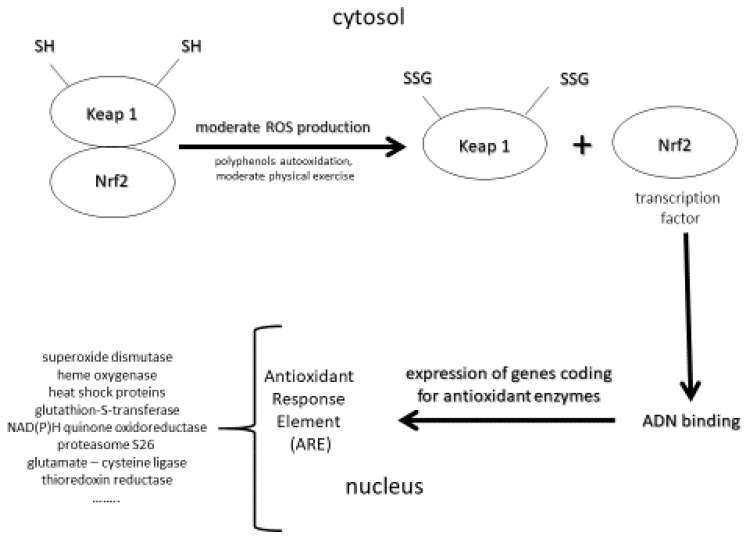
Principle of hormesis. Upon contact with moderate amounts of ROS, Keap1 and Nrf2 proteins separate following the oxidation of the thiol groups (-SH) present on the Keap1 protein. Nrf2 becoming a transcription factor then migrates into the cytosol where it binds to DNA. The result is the activation of genes coding for a large number of antioxidant enzymes (Antioxidant Response Element (ARE)).

It is well accepted that oxidative stress (OS) is implicated in the development of many human pathologies. Initially, pathological OS has been defined as an imbalance between ROS (free radicals, hydrogen peroxide, and singlet oxygen) and the antioxidant network in favor of the former, leading to oxidative damage to lipids, more particularly polyunsaturated fatty acids, DNA as evidenced by the measurement of 8-hydroxy-2′-deoxyguanosine (8-OHdG) or its oxidized form—8-oxo-7,8-dihydro-2′-deoxyguanosine (8-oxodG)—and proteins [22,23] implicated in the development of several human pathologies, such as cardiovascular diseases and atherosclerosis [24], and all cancer types, including skin [25] and neurological disorders such as Alzheimer’s disease [26]. However, molecular biology has highlighted that physiological ROS, more specifically, hydrogen peroxide, control several normal functions at the cellular level [27,28]. Indeed, physiological ROS can act as secondary messengers, leading to the activation of important protective mechanisms for our body (e.g., apoptosis, p-53DNA reparation) [29]. To reconcile both deleterious and physiological aspects of ROS, Sies and Jones [30] defined OS as an imbalance between reactive oxidative species or ROS and antioxidants in favor of the formers, leading to a signaling disruption as a consequence of oxidative damages to lipids, and DNA evidenced by the measurement of proteins. A third interesting and novel concept is adaptive oxidative stress or hormesis (Figure 1) defined as a phenomenon in which ROS produced in moderate amounts had beneficial effects to living organisms [31,32,33].

**Figure 2:**
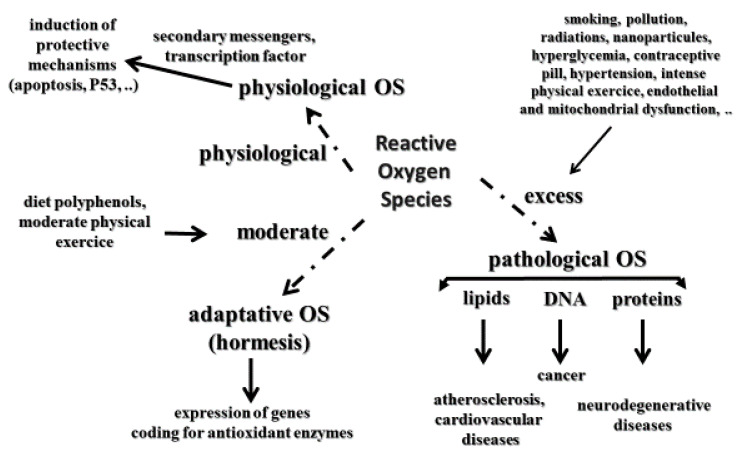
The three types of oxidative stress (physiological, pathological and adaptative) according to the amount (physiological, excess, and moderate) of ROS production and their biological roles.

Therefore, these important notions of OS must be well integrated within the framework of effects provided by antioxidant supplements taken orally (Figure 2). Indeed, the role of physiological OS can in no way be neglected, especially in the context of antioxidant supplementation at supra nutritional and even nutritional doses in some cases. If the redox balance is disrupted by excessive antioxidant consumption taken for long periods, this may be the cause of increased cancers [34,35].

## 3. Oxidative Stress and Skin

Human skin is made up of the epidermis on the surface and the dermis below. The hypodermis is located between the epidermis and the hypodermis. The epidermis, which is 90%–95% keratinocytes, is made up of five layers, including the stratum basale (the deepest portion of the epidermis), stratum spinosum, stratum granulosum, stratum lucidum, and stratum corneum or SC (the most superficial portion of the epidermis). Sebum is a lipid film secreted by the sebaceous glands of the skin, which lubricates the skin and helps to retain moisture. Skin is the main interface between the body and the environment, providing a biological barrier against chemical and physical pollutants. Having the largest surface area of the body, skin is the major organ target for increased oxidative stress resulting in its alteration of the antioxidant network and, consequently skin aging and diseases [36,37,38,39,40]. As the skin ages, the number of keratinocytes and fibroblasts decreased as a consequent reduction in the turnover of the epidermis and the subsequent decrease in collagen and proteoglycans. These changes may lead to a further increase in the production of free radicals. All this leads to atrophy and contributes to fibrosis [41].

Intrinsic (chronological age) and extrinsic factors contribute to enhanced OS in the human skin, which induce and accelerate the skin aging process [6,7,8]. Internal aggressions are due to food contaminants, mitochondrial dysfunction in keratinocytes and fibroblasts, activation of lipoxygenase and cyclooxygenase in the cytosol, NADPH oxidases, cytochrome P450 located in the endoplasmic reticulum, inflammation via overexpression of activator protein-1 (AP-1) and the nuclear factor kappa B (NF-κB) [42]. These aggressions are associated in the dermis with a loss of collagen and elasticity, skin atrophy, wrinkles, degeneration in the elastic fiber network and loss of hydration [43]. External aggressions result from ozone exposure, pollution generated by automobiles and other industrial sources, smoking, excessive alcohol consumption, professional or personal stress, insomnia, cosmetic products, chemical products, pathogens, sedentary lifestyle and an unbalance diet [42,44]. These aggressions induce rough texture, irregular pigmentation and deep wrinkles [45]. Both intrinsic and extrinsic OS also contribute to the development of most common skin diseases such as acne, alopecia areata, atopic dermatitis (eczema), psoriasis, Raynaud’s phenomenon, rosacea, vitiligo, and skin cancer [46].

An important ROS external source is photo-aging (dermatoheliosis) as the result of a continuous, long-term exposure to ultraviolet A and B (UVA and UVB) radiation of approximately 300–400 nm, either natural or synthetic, on an intrinsically aged skin [47]. Direct proof of free radical production during UV-exposed animal and human skin has been assessed using different detection methods [48,49,50,51,52]. Photoaging is characterized by the appearance of wrinkles, dark spots, skin thickening, wrinkling, and certain lesions such as actinic keratosis and cancer. Exposure also leads to inactivation and loss of epidermal Langerhans cells, which are an important part of the skin’s immune system. Other than large ROS production, excessive exposure to UV radiation also induces inflammation, immunosuppression, induction of metalloproteases, DNA oxidative damage and skin disorders such as psoriasis [16,53].

A high amount of ROS produced in the skin quickly overwhelms skin antioxidant defense, and consequently, induces important oxidative damages to lipids (lipid peroxides, large protein-lipid aggregates known as lipofuscin), DNA (8-hydroxy-2′ -deoxyguanosine or 8-OHdG) and proteins (protein carbonyls). Squalene (2,6,10,15,19,23-hexamethyltetracosa-2,6,10, 14,18,22-hexaene), cholesterol and sebaceous acids being the main components of sebum and skin surface lipids are an important target for singlet oxygen oxidation [54]. As shown in Figure 1, polyunsaturated fatty acids, a main constituent of cellular membrane, are also very sensitive to ROS attack resulting in lipid peroxidation process by auto-oxidation, which is amplified by transition metals such as iron or copper. As reported by Bickers and Athar [38], oxidation of skin surface lipids may be important in the appearance of wrinkle formation, hyperpigmentation, freckles, acne, atopic dermatitis, and cancer. Sanders et al. [55] evidenced increased proteins carbonyl as a marker of protein oxidation in the skin of healthy subjects irradiated with acute UVA exposure. Moreover, photoaged skin also revealed a significantly lower expression of important antioxidant enzymes including superoxide dismutases and catalase in the epidermis and SC. In physiological conditions, guanosine pairs with cytosine. In contrast, oxidized guanosine (8-OHdG) pairs with adenosine, resulting in a G-T mutation that accumulates during aging in skin [56]. A dose-response for induction of primary skin fibroblasts (FEK4) by broad-band UVA (350–450 nm) radiation has been reported by Kvam and Tyrell [57]. All these oxidative damages are aggravated by the accumulation of labile or free iron, a strong catalyst of ROS production through the Fenton reaction, during skin aging [58]. Moreover, exposure to UVA radiation contributes to the degradation of ferritin in the skin and that causes the release of labile iron leading to the appearance of oxidative damages due to the imbalanced redox status in the cell. In addition to altering DNA, proteins and the cell membrane increased ROS production promotes matrix metalloproteinase (MMP) resulting in collagen breakdown [59].

There is also ample evidence that glucose and advanced glycation end products (AGEs) play an important role in skin aging [60,61,62,63]. Irreversible AGEs are formed in vivo through three different pathways: The Maillard reaction resulting from the interaction between a carbonyl group of a reducing sugar with the amino group of a protein leading to unstable Amadori products, the polyol pathway leading to the conversion of glucose into fructose being further converted to 3-deoxyglucose and finally the reaction of lipid peroxidation byproducts with dicarbonyl proteins [64,65]. AGEs can cause the crosslinking of mitochondrial proteins in the respiratory chain, reducing the synthesis of ATP and promoting the production of oxidative free radicals [66,67]. AGEs have been shown to be increased in the epidermis as well as photoaged and diabetic dermis [68,69]. In both in vitro experiments [70] and in vivo [71] studies, it has been shown that UV irradiation may also enhance the formation of AGEs in the skin. A diet too high in sugar (hyperglycemia) and certain methods of food preparation are also responsible for higher levels of AGEs in the skin [72]. Interestingly, a non-invasive method (AGE-Reader, DiagnOptics B.V., Groningen, The Netherlands) has been developed to measure the skin content of AGEs. It was shown that skin autofluorescence increases with chronological aging and correlates with skin deposition of AGEs, making this method a potential tool for investigating the effect of various anti-aging products in the cosmetic industry [73]. AGEs not only exert their deleterious actions due to their biological properties, but also through their interaction with specific receptors for advanced glycation end products (RAGE) that can directly induce oxidative stress [74,75] and inflammation in skin. Through the alteration of the physicochemical properties of dermal proteins, decreased cell proliferation, increased apoptosis and senescence, induction of oxidative stress and proinflammatory mediators, as well as other pathways, the AGEs/RAGE axis contributes to skin aging.

Another important target for ROS is telomeres that protect the ends of chromosomes from degradation and from being recognized as double-stranded breaks [76]. The maintenance of telomere length appears to play a fundamental role to delay the aging process [77]. Due to the high G content in telomeric structures, oxidative stress accelerates the telomere attrition, a leading cause of skin aging [78,79,80,81,82]. In 2011, Buckingham and Klingelhutz [83] described in detail how the interplay between oxidative stress, DNA damaging, and accelerated telomere shortening plays a key in the aging of human skin. Sugimoto et al. [84] also reported the association between telomere length of the skin with chronical aging and photoaging confirming previous observations done by Kosmadaki and Gilchres [85]. Jacczak et al. [86] claimed that maintaining the key levels of telomerase component (hTERT) expression and telomerase activity that provide optimal telomere length, as well as some non-telomeric functions, represents a promising step in advanced anti-aging strategies, especially in dermocosmetics.

**Figure 3:**
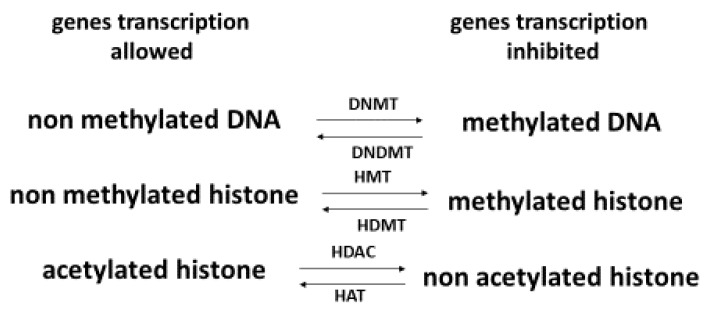
Effect of DNA methylation and histone methylation and acetylation on gene expression (epigenetics). DNMT: DNA metyltransferase, DNDMT: DNA demetyltransferase, HMT: histone metyltransferase, HDMT: histone demethyltransferase, HDAC: histone deacetylase, HAT: histone acetyltransferase.

A key factor in health and skin aging is epigenetics [87,88], which refers to external modifications to DNA that turn genes “on” or “off.” These modifications, which do not change the DNA sequence, allow cells to express or not specific genes that are necessary for the existence of different cell types. As shown in Figure 3, epigenetics intervenes at diverse levels: DNA methylation, histone methylation and histone acetylation [89]. Histones are important to help condense DNA into chromatin. Hyper DNA and histone methylation lead to a low expression of genes, while non acetylated histone hinders gene expression. In older individuals, Johnson et al. [90] showed that their DNA was characterized by a specific hypermethylation pattern. In the context of skin aging, Russell-Goldman and Murphy [91] reported that modification of epigenetic factors may result in deterioration of the main protective interface of the skin with the external environment. Köhler et al. [92] showed that young epidermis was characterized by unmethylated CpG in contrast to old epidermis. de Oliveira et al. [93] reported that UV radiation can alter the DNA methylation profile in epidermal cells derived from the skin. Recently, Boroni et al. [94] developed a highly accurate skin-specific DNA methylation age predictor. Based on the analysis of 2266 CpG sites, the authors showed that DNA methylation (DNAm) age was sensitive to the biological age of the donor, skin disease status, as well as treatment with senotherapeutic drugs. Recently, Orioli and Dellambra [95] reviewed the important role of epigenetics in skin cells in relation to natural aging and premature aging diseases. The authors highlighted that in patients with progeroid syndromes characterized by an accelerated aging process in various organs, including skin, accumulation of DNA damage, and increased genome instability, but also epigenetic changes have been evidenced in their skin cells.

## 4. The Skin Antioxidant Network

In order to counteract the deleterious effects of ROS, human skin is equipped with a network of antioxidants, including enzymes and low molecular weight molecules [96]. The main enzymes, which represent the first line of defense against ROS, are superoxide dismutases (SODs), catalase, glutathione peroxidases (GPxs), glutathione-S-transferase, thioredoxin reductase, and many others. Their activity is dependent on some trace elements, such as copper and zinc (SOD) or selenium (GPx). The second line is composed of low molecular weight compounds, including vitamins C and E, carotenoids, glutathione, ubiquinone, lipoic acid, uric acid, polyphenols and melatonin. This last compound has the particularity to be synthesized in the skin [97] and dotted of antioxidant and anti-inflammatory activities [98,99]. Through mechanisms involving free radicals, melatonin is metabolized in skin into metabolites N1-Acetyl-5-methoxykynuramine (AMK) and N1-acetyl-N2-formyl-5-methoxykynuramine (AFMK) having a much higher antioxidant capacity [100]. Melatonin has also been shown to be an activator of the Keap1-NrF2 antioxidant pathway in the skin, but only through topical application [99].

Scarce data are available regarding the antioxidant concentration in the skin. The best information was obtained as early as 1994 by Shindo et al. [101], showing that the human striatum corneum exhibits antioxidant capacity with logical increasing intensity from the dermis to the epidermis, the last being more exposed to the external environment. Activities of superoxide dismutase, catalase, glutathione peroxidase, and glutathione reductase enzymes were largely higher in the epidermis compared to the dermis. The vitamin E (alpha- and gamma-tocopherol) level was 34.2 nmol/g skin compared to only 18.0 nmol/g in the dermis. A difference of 425% and 488%, respectively, was found for vitamin C and uric acid levels between epidermis and dermis (3798 vs. 723 nmol/g skin; 1071 vs. 182 nmol/g skin). Content of reduced glutathione was 461 nmol/g skin in the epidermis versus 75.1 in the dermis. The level of ubiquinone was at least 900% higher in the epidermis than in the dermis (3.53 nmol/g skin vs. 0.35). Notably, the regional differences in vitamin E levels, the human facial stratum corneum, which is continuously exposed to environment, contains higher vitamin E levels than the less exposed upper arm [102].

It has been recognized for a long time that natural antioxidants, such as vitamins C and E, play a key role in skin care exerting different actions including photoprotection, increased antioxidant activity, collagen synthesis and keratinization [103]. In contrast, blood antioxidant deficiency has been associated with many skin alterations [104]. For example, vitamin C deficiency (RDI = 100 mg) is well-known to cause scurvy, a disease with skin lesions, including petechiae, gum bleeding, ease of developing bruises or slow wound healing. Cutaneous manifestations accompanying vitamin C deficiency have been attributed to impaired collagen synthesis [105]. Enlargement and keratosis of hair follicles, mainly in the upper arms and curled hairs, the so-called ‘corkscrew hairs’, are also usually described. Therefore, nutrition, which constitutes the natural way to bring important antioxidants, such as vitamins C and E, carotenoids, and ubiquinone, is of primordial importance for skin health [10,106,107]. In this way, the Mediterranean diet, which contains many bioactive nutrients (vitamins C and E, polyphenols, carotenoids, etc.) has been shown to be effective in providing internal protection against UVR [108]. In a 15-year study period performed on 777 subjects, adults aged >45 years who ate foods with high antioxidant capacity exhibited approximately 10% less photoaging over 15 years than those who consumed foods with low antioxidant capacity [109].

Exposure to UV contributes to a significant depletion in skin enzymatic and non-enzymatic antioxidants [110,111,112]. Rhie et al. [113] reported significant low α-tocopherol levels in the epidermis of photoaged skin but not in the dermis. Vitamin C and glutathione levels were lower in the dermis and epidermis of photoaged and naturally aged skin by contrast to uric acid. Interestingly, UV exposures given for 11–12 days (cumulative doses of UVA 17.8 ± 1.9 J/cm^2^) in humans resulted in a significant decrease in plasma β-carotene [114]. Biesalski et al. [115] also reported a similar observation in addition to a depletion in vitamin E after 12 days of controlled sun exposure (total UV dose 10.000 j/cm^2^).

## 5. Selenium

### 5.1. Physiological Functions

Selenium (Se) is a natural mineral obtained from food including cereals, breads, millets, wheat (6.7 oz = 171 µg), Brazil nuts (6 to 8 nuts = 544 µg), meats (3 oz turkey = 32 µg, 3 oz chicken = 20 µg), eggs (one boiled egg = 14 µg) and mushrooms (3.5 oz = 12 µg) [116,117,118]. Many papers have highlighted the role of Se in human health [119,120,121,122,123]. In living organisms, Se is present in various organic forms including selenocysteine (SeCyS) as a predominant form. It is specifically incorporated into seleno proteins, including six glutathione peroxidases (GPxs), three deiodinases (Dis), 12 selenoproteins (H, I, K, M, N, O, P (SePP1 or SELENOP), R, S, T, V, W, and three thioredoxin reductases (TrxR1, TRxR2, and TRxR3) [124,125].

Selenoprotein-mediated biochemical mechanisms (regulation of oxidative stress, antioxidant defense, immune and inflammatory responses and other biological processes play a key role in the prevention, onset, and clinical outcome of a wide number of important diseases (cancers, viral infections, mental disorders, diabetes, etc.) [117,126,127]. A special focus should be given to SELENOP. Besides being the main selenium-transporter, this protein plays a central role in selenium-metabolism and antioxidant defense by maintaining the antioxidant capacity of selenoenzymes [128]. TrxRs play a fundamental role in the regulation of the homeostasis of protein thiol and ROS signaling, both in the intra- and extra-cellular milieu [129]. GPx isoenzymes reduce hydrogen peroxide (H_2_O_2_), organic hydroperoxides, and (only GPx4) phospholipid hydroperoxides, using reduced glutathione (GSH) as co-substrate according to the following equations [130]:

(1) $$H_{2}O_{2}+2\mathrm{GSH}\overset{\mathrm{GPx}}{\to }H_{2}O+\mathrm{GSSG}$$

(2) $$\mathrm{ROOH}+2\mathrm{GSH}\overset{\mathrm{GPx}}{\to }\mathrm{ROH}+\mathrm{GSSG}+H_{2}O$$

A minimum of 55 µg Se/d seems to be required to maximize the activity of glutathione peroxidases (GPx1 and GPx3) in order to maintain their antioxidant function at the possibly highest level [131]. Around 100 µg Se/d effectively saturate selenoprotein P that is used specifically to assess the body’s Se status [132,133].

It is well-known that Se deficiency is associated with health risks in humans [119]. By contrast, an adequate Se nutritional state has been related to improved outcomes and reduced risk of developing several diseases [120]. In the case of a low plasma Se concentration (<89 µg/L), it is appropriate to increase Se intake through diet or a modest Se supplementation (50–100 µg/d) in order to reach an ideal plasma Se <122 µg/L [119,134]. Symptoms of Se deficiency include skin damage, weakening of immune defenses, muscular, neurological, and cardiovascular disorders, Kashin-Beck disease, and developmental abnormality. The tolerable upper intake limit is 300–400 μg/d for adults over 19 years old, a value below standard Americans of 400 μg/day. Chronic Se intake at >900 µg is toxic due to selenosis development [135]. Signs of Se toxicity include hair loss, dizziness, nausea, vomiting, facial flushing, tremors, muscle soreness, severe fatigue, irritability, bad breath, sensitivity to inflammation, stained fingernails, mottling of teeth, nerve lesions and diarrhea. In severe cases, acute Se toxicity can lead to serious intestinal and neurological symptoms, heart attack, kidney failure, and death.

### 5.2. Selenium, Skin Aging and Disorders

In 2000, McKenzie [136] reviewed the potential effect of Se to protect skin from damages induced by UV radiation. In cultured cells, both selenomethionine (SM) and sodium selenite (SS) at nanomolar concentrations can protect keratinocytes, melanocytes and fibroblasts from UV-induced cell death, accumulation of 8 OHdG as a marker of DNA oxidation and apoptosis. UVB-induced accumulation of lipid peroxides in keratinocytes is decreased by 82% if the cells are pre-treated with 50 nm SM before irradiation with 200 J/m^2^ UVB. In a senescent skin equivalent model, Jobeili et al. [137] showed that SS in a concentration range from 0.633 to 1.875 µM preserved kerotinocytes and delayed senescence by maintaining epidermal adhesion. In cultured human skin cells, 10 nM SS or 50 nM SM were able to reduce the lethal effects of UVB radiation on primary keratinocytes by 57.3% and 65.8% respectively [138]. In keratinocytes isolated from young donors, low doses of Se (30 nM) protect cells against UVA-induced cytotoxicity, whereas the protection efficiency of Se in old keratinocytes required higher concentrations (240 nM) [139]. Moreover, the same group showed that Se supplementation significantly enhanced the DNA repair of 8-oxoguanine (8oxoG) only in the keratinocytes isolated from young donors. In a population of 8 women treated for 2 weeks with a topical application of SM, the UVB dose necessary to achieve 1 minimal erythema was increased by 30% [140]. In a recent paper, Alehagen et al. [141] reviewed all potential mechanisms by which Se may have a positive impact on aging and potentially on skin aging. As explained below, Se is fully implicated in the activity of these selenoproteins such as GPx, TRxRS and SeH which are essential for cell survival [127,129,130]. Indeed, these enzymes play a key role in cellular redox regulation by reducing ROS and thus lipid and DNA oxidation.

We have shown that below this oxidative stress is associated with telomere shortening and the aging of human skin [83]. Se contributes to prolonged telomere length [120]. An observational study on 3194 Americans older than 45 years showed that every increase of 20 µg/Se intake was associated with 0.42% longer telomere length in all participants [142]. In their paper, Jacczak et al. [86] concluded that natural compounds, such as vitamin E, polyphenols, and Se, able to provide optimal telomere length could represent advanced anti-aging skin strategies. Another major actor of skin aging is the accumulation of advanced glycation end products [63]. In a dose-dependent, response, sodium selenite and Se yeast are able to significantly inhibit the bovine serum albumin glycation induced in vitro by exposure to glucose/fructose [143,144]. Recently, Du et al. [145] also showed that Se nanoparticles (SeNPs) have certain inhibition ability against glycation. A particularly important role of Se is its relationship with epigenetics.

In 2015, Speckmann and Tilman [146] discussed the relevance of a Se-epigenome interaction for human health. The authors listed fifteen articles showing that Se is able to reduce DNA methylation by inhibiting DNMT expression in various types of cells. Inhibition of histone deacetylases. Interestingly an inverse association between plasma Se and leukocyte DNA methylation [147]. Moreover, inhibition of histone deacetylase (HDAC) delay has been shown in experimental models to delay aging by maintaining genome stability [148]. Histone modifications have been shown to be altered by Se via the inhibition of HDAC activity by the Se metabolism products seleno-ketoacids such as methylselenopyruvate and α-keto-γ-metylselenobutyrate [149]. Recent papers have shown that proteins from the sirtuin (silencing information regulator) family (SIRT1-7) belonging to the class III histone deacetylases were strongly linked to epigenetic regulation and therefore genome stability [150]. These proteins can regulate many processes in vivo, including DNA repair, prevention of telomere attrition and metastasis, decreased oxidative stress, promotion of longevity, and the protective effect against cancer [151]. Their biological relevance in the regulation of aging, and age-related diseases has been recently reviewed by Zhao et al. [152]. By sustaining genome integrity, sirtuins are now considered to be promising therapeutic targets for anti-aging skin and related diseases [153,154,155]. Of interest is that low serum Se (<0.75 µM) is associated with the downregulation of sirtuin concentration in peripheral blood mononuclear cells [156].

### 5.3. Se Plasma Concentration, Skin Disorders and Supplementation

A study on the different European countries reported values of mean intake in adults of 43 µg/d corresponding to normal plasma values between 70 and 120 μg Se/L [157]. A large consensus defined plasma Se concentrations from 70 to 100 μg/L (0.9 to 1.3 μmol/L) to reflect Se adequacy [158]. Se supplements usually come in three forms: selenite, selenomethionine and high-selenium yeast (yeast). Burk et al. [159] showed that plasma Se concentration were markedly increased with the two last forms when compared to selenite. selenite. When compared to placebo, supplementation with 158 µg/d selenomethionine for four weeks resulted in plasma Se concentration increase from 121 to 215 µg/L. Such a plasma value did not significantly increase after 16 weeks supplementation. At a higher dose of 507 µg/d, plasma concentration rose to 336 µg/L after four weeks. Supplementation with 226 µg/d and 703 µg/d yeast caused, respectively, plasma concentration to increase up to 176 µg/L and 341 µg/L. Prolonged supplementation to 16 weeks did not change these plasma values.

In a meta-analysis of twenty-seven studies, including a total of 1315 patients and 7181 healthy controls, lower plasma Se level was found in patients with psoriasis, acne vulgaris, chloric acne and atopic dermatitis when compared to the control [160]. By contrast, no significant difference in Se was found in patients with vitiligo, alopecia areata and eczema. In older studies, low levels of blood Se-dependent GPx were observed in patients with psoriasis, eczema, atopic dermatitis, vasculitis, mycosis fungoides and dermatitis herpetiformis when compared to the control population [161]. In a recent systematic review, Vaughn et al. [162] demonstrated that plasma Se deficiency may exacerbate atopic dermatitis (AD). Using hair samples, analyzed by proton induced X-ray emission (PIXE) for 32 mineral concentrations during one and ten-month health checkups, Yamada et al. [163] confirmed that Se deficiency significantly increased the AD risk in infants. In 29 acne vulgaris patients, supplementation with 0.2 mg Se (and 10 mg tocopheryl succinate) for 6–8 weeks slowly increased Se-GPx levels [164]. In contrast, if the low levels of Se glutathione-peroxidase in blood from patients with dermatitis herpetiformis increased after five months of Se treatment, no significant clinical improvement was observed [165]. Fairris et al. [166] showed that a daily 600 µg of Se-enriched yeast intake for 12 weeks increased plasma Se concentrations and platelet GPx activity of psoriasis patients, but not in their skin. No reduction of psoriasis severity was observed.

## 6. Vitamin E

### 6.1. Physiological Function

Vitamin E is the collective name for a group of eight fat-soluble compounds, including four tocopherols (α-, β-, γ-, and δ-tocopherol) and four tocotrienols (α-, β-, γ-, and δ-tocotrienol). Since vitamin E is fat-soluble, it is mainly found in an amount between 1.3 to 20.3 mg/serving in vegetable oils (soya, corn, sunflower), but also in oleaginous fruits (walnuts, hazelnuts, and almonds) and whole grain germs [116]. The most abundant forms of vitamin E in the diet are α-tocopherol and γ-tocopherol. Current nutrient reference value (NRV) for vitamin E as α-tocopherol is 12 mg/d (17.6 IU (International Unit) and 26.64 IU, respectively, for natural and synthetic forms). The tolerable upper intake level was fixed at 300 mg/d by the European Food Safety Authority [167]. However, high doses of vitamin E may increase the risk of bleeding, particularly for adults who are also taking an anticoagulant (especially warfarin), which makes blood less likely to clot. Occasionally, adults who take very high doses develop muscle weakness, fatigue, nausea, and diarrhea [167].

**Figure 4:**
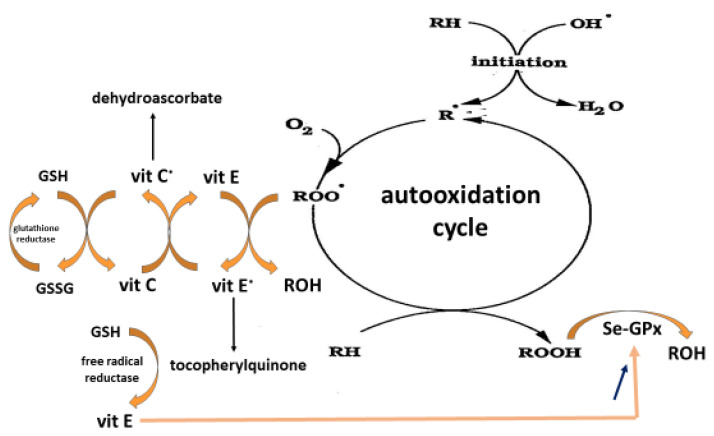
Lipid peroxidation process (autoxidation of polyunsaturated fatty acids (RH)). See the explanation in the text.

As shown in Figure 4, vitamin E (Vit E) as α-tocopherol functions as a chain-breaking antioxidant inhibiting the free radical-chain peroxidation of polyunsaturated lipids in membranes and lipoproteins. Free fatty acids (RH), more specifically, the polyunsaturated ones (ω3 and ω6), are extremely sensitive to oxidative damage induced by ROS, such as hydroxyl radical (OH^•^) In an initiation step, RH becomes a free radical species (R^•^) that interacts with oxygen (O_2_), leading to the formation of a new free radical species ROO^•^ (peroxyl radical). This then interacts with a neighboring free fatty acid (RH) to finally generate a toxic lipid peroxide (ROOH) and another lipid radical R^•^, thereby initiating an auto-oxidation cycle. Vitamin E (consisting of various forms of tocopherols and tocotrienols) acts as an important fat-soluble vitamin in strong synergy with vitamin C and reduced glutathione (GSH) to break the chain. Indeed, Vit E can directly interact with ROO^•^, resulting in the formation of a stable and nontoxic lipid (ROH). However, Vit E becomes a free radical species (vit E^•^ or tocopheryl radical) which becomes neutralized by vitamin C (vit C). This last antioxidant itself becomes a free radical (vit C^•^ or ascorbyl radical). Finally, glutathione (GSH), which regenerates the ascorbyl radical in its initial form, is converted into oxidized glutathione (GSSG), thereby ending the peroxidation cycle [168,169,170]. These mechanisms demonstrate that the low molecular weight antioxidants do not act in isolation but form an intricate network. This therefore requires a good equilibrium between them to break the lipid peroxidation process. Interestingly, vitamin E is also able to increase the human seleno-GPx activity, which is involved in lipid peroxide destruction [171,172,173].

### 6.2. Vitamin E, Skin Aging and Disorders

Vitamin E is the major naturally occurring lipid-soluble non-enzymatic antioxidant protecting skin from the adverse effects of oxidative stress [174]. In animal models, vitamin E deficiency resulted in skin ulcerations and changes in skin collagen cross-linking [175]. In humans, deficiency in vitamin E from the diet has been shown to also cause skin anomalies [176,177]. With respect to photoaging, Thiele et al. [96] showed that exposure to a single dose (0.75 MED) of solar simulated ultraviolet light dose dependently depleted the SC concentrations of α-tocopherol in mice by 85%. Such depletion was later confirmed by Lester and Valacchi [178], Fryer et al. [179], Nachbar and Korting [180] and Pandel et al. [111]. In agreement with Rhie et al. [113], Tiele et al. [181] also reported a significant depletion of tocopherols (α-tocopherol by 45%, and γ-tocopherol by 35%) in human SC exposed to a single suberythemogenic dose of solar simulated UVR as compared to the controls.

Using a topical application, the antioxidant properties of vitamin E have been well evidenced [182,183,184]. Besides antioxidant activity, vitamin E in topical application may also exert skin protective effects through anti-inflammatory properties [185,186,187] and its ability to inhibit the action of metalloproteinases [188]. Vitamin E may also interfere with global DNA methylation [189]. Mackpol et al. [190] also described the ability of tocotrienol-rich fraction in preventing cellular senescence human diploid fibroblasts (HDFs) by restoring telomere length and telomerase activity, reducing the damaged DNA, and reversing cell cycle arrest associated with senescence. Vitamin E is also able to modulate the protein kinase C (PKC) and phosphatidylinositol 3-kinase (PI3-K) signaling pathways and to reduce the increase in collagenase expression in skin [191]. The photo protective effects of vitamin E in topical application against skin damage induced by UV exposure results in reducing skin roughness, skin dehydration, elastosis, wrinkling, facial lines and senile lentigines [192].

In contrast, Table S1 (Supplementary) listed the major but limited studies having investigated the photo protective effect of vitamin E taken by an oral route. The study performed by La Ruche and Cesarini [193] evidenced that a daily nutritional dose of 14 mg vitamin E (31 IU) but associated with retinol and selenium for three weeks was able to reduce the number of skin sunburn cells after UV exposure. Due to a very small subject number (*n* = 16), these results have, however, to be taken with caution. In other studies [194,195,196], except one [197], supra nutritional doses from 50 to 2000 mg/d (110 to 4428 UI vitamin E) taken over a period of 50 days to eight weeks were able to improve the minimal erythema dose (MED). Supra nutritional doses were also necessary to observe a clinical improvement in skin ageing and disorders such as atopic dermatitis, acne, psoriaris or vitiligo [198,199,200,201,202,203,204,205,206], see also the work by Berardesca et al. [207]. Table S2 (Supplementary) displays human studies using a combination of vitamin E with other antioxidants and/or Se in skin photoprotection and diseases [166,194,208,209,210,211,212,213,214,215]. Some studies [194,195,208,209,212] showed the photoprotection of vitamin E was amplified in the presence of vitamin C taken at supra-nutritional doses (2 to 3 g). This is in agreement with their synergistic action, as described in Figure 1 [216].

### 6.3. Vitamin E Plasma Concentration, Skin Disorders and Supplementation

The reference values for plasma vitamin E (α-tocopherol) are 8.60–19.2 µg/mL and 4.40–7.00 µg/g after standardization to cholesterol [217]. Roberts et al. [218] examined the relationship between dose of vitamin E (supplementation in a range of 45 mg (100 IU) to 1454 mg (3200 IU)/d) taken for 16 weeks and suppression of oxidative stress, as measured by plasma isoprostanes. After 16 weeks, initial plasma vitamin E increased by 25% at a dose of 45 mg/d up to 140% at a dose of 727 mg/d. Moreover, there was a linear trend between the dosage of vitamin E and percent reduction in plasma F2-isoprostane concentrations, which only reached significance at doses of 727 mg/d (35% ± 2%, *p* < 0.035) and 1454 mg/d (49% ± 10%, *p* < 0.005) in vitamin E. Biesalski et al. [115] reported that plasma α-tocopherol in humans was significantly decreased after exposure to sunlight for 12 days (total UV dose of about 10.000 J/cm2). In a systematic review and a meta-analysis screening of 892 studies, Liu et al. [176] reported lower serum vitamin E levels in vitiligo, psoriasis, atopic dermatitis, and acne patients when compared to the controls. As an example, Ozuguz et al. [219] found a significant decrease in the serum vitamin E concentration of patients with acne vulgaris when compared to controls (7.88 ± 3.00 µg/mL vs. 11.06 ± 3.0 µg/mL, *p* < 0.001). In Seborrheic dermatitis (SD), a common form of skin disorder, Jahan et al. [104] recently reported lower plasma levels in vitamin E 5.54 ± 0.79 µM vs. 7.07 ± 0.37 µM, *p* = 0.009) when compared to the controls. In contrast, increased concentration in malonaldehyde (MDA), as a marker of lipid peroxidation, (0.21 ± 0.18 µM vs. 0.18 ± 0.28, *p* = 0.011) was detected in parallel with higher levels in copper (2.13 ± 0.10 mg/L vs. 0.95 ± 0.05 mg/L, *p* < 0.001) known to catalyze the free radical reaction.

## 7. Oxidative Stress, Antioxidants, and Aging Hair

Similar to the skin, the scalp is also subject to ageing, which manifests as a decrease in the mel-anocyte function or graying, and a decrease in hair production or alopecia. As reported by Trüeb [220,221,222], the gradual loss of pigmentation includes exhaustion of the enzymes involved in melanogenesis, impaired DNA repair, loss of telomerase, and antiapoptotic signals, but also increased oxidative stress as a pivotal role. Androgenetic alopecia (AGA) is a genetically predetermined disorder due to an excessive response to androgens and environmental factors. In males, hair loss is the most prominent in the vertex and frontotemporal regions, while in women, the frontal hairline is typically spared with diffuse hair loss at the crown and top of head, with loss often marked by a wider center part [223]. The presence of inflammation and of oxidative stress in both dermal papilla cells and plasma of AGA patients have been evidenced [224,225].

Alopecia areata (AA) is an inflammatory and autoimmune disease presenting with non-scarring hair loss. Prie at al [226] reported that many factors, such as autoimmunity, genetic predisposition, emotional and environmental stress, all processes having in common increased OS, were thought to play important roles in AA development. In a recent meta-analysis of 18 studies, Acharya and Mathur [227] evidenced elevated markers of lipid peroxidation and a decrease in antioxidants in the plasma of AA patients. With respect to plasma depletion in vitamin E, the results were contrasting [228]. One study on 15 AA patients reported a depletion [229], and another on 37 patients showed different results. [230]. Thompson et al. [231] reviewed the role of trace elements in AA. Zinc was found to be depleted in AA patients whereas results seem to more conflicting for Se. Nevertheless, El-Tahlavi et al. [232] reported that plasma Se concentration was found to be significantly decreased in AA patients when compared to the controls (60.2 ± 8.8 µg/L vs. 77.4 ± 9.6 µg/L).

In the context of SARS-CoV-2 infection, it has been recently reported AGA may be a risk factor for severe COVID-19, whereas telogen effluvium (TE) presents as a sequela of COVID-19 [233,234,235]. In our recent studies [236,237], we have shown that 55.5% COVID-19 patients hospitalized in intensive care unit, 87.5% in ward units or 58.3% seen two months after their hospital discharge exhibited Se plasma concentration below normal range (73–110 µg/L). Other papers further confirmed the presence of an important Se depletion in COVID-19 patients [238,239]. In their paper, Guo et al. [240] concluded that there is limited research on Se deficiency and AA in humans. However, the association between low plasma Se levels, AA and SARS-CoV-19 infection may suggest that a Se supplementation would be useful in COVID-19 patients, not only to reduce the severity of the pathology [241], but also to reduce sequela, such hair loss associated with the disease. It is important to keep in mind that Se taken in excess causes hair loss [135]. In the biology of hair follicle, we must highlight the importance of preserving the telomere length against OS, as shown in experiments on epidermal stem cells [242,243]. It has been explained below how Se and vitamin E might slow down the telomere attrition.

Topical application with vitamin E oil, acting as an antioxidant, has been reported to prevent premature aging, expand the capillaries, resulting in an increased blood flow in the scalp, and moisturize the hair [244]. There are however very limited data about the effect of vitamin E supplementation. Only one study [245] has shown that a mixture of tocotrienols taken every day for 4–8 weeks was able to increase the hair number in AA patients. By contrast, another study has reported the adverse effects on hair growth in volunteers taking every day excessive vitamin E (270 mg or 600 IU) during only 28 days [246].

## 8. Synergic Antioxidant Action of Vitamin E/Se

Using Cyclic Voltammetry (CV) and Osteryoung Square Wave Voltammetry (OSWV), Bertolino et al. [247] have shown, in a very good in vitro study, the existence of an important synergism between Se and some other natural and synthetic antioxidants such as vitamin E. Alone, vitamin E had the highest antioxidant efficacy (AE) to scavenge DPPH (2,2-Diphenyl-1-picrylhydrazyl) radical. In the presence of Se, AE increased from 84.5 10^−3^ up to 315.2 10^−3^.

**Figure 5:**
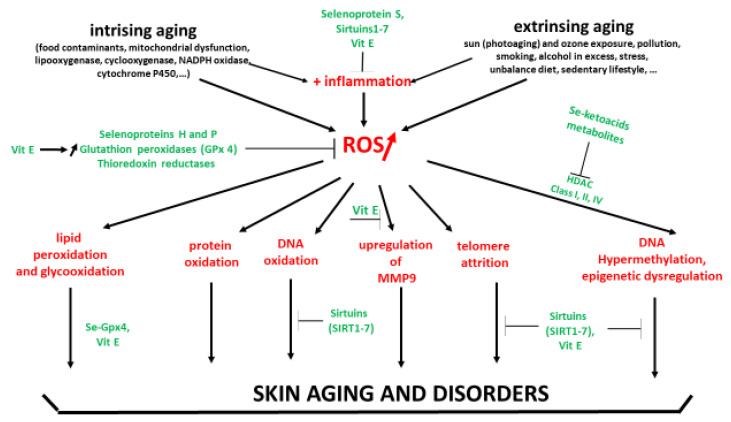
Se and vitamin E: Complementary mechanisms of action against skin aging and disorders. Adapted with permission from Alehagen et al. [141]. Red: pro-oxidant sources; green: antioxidant defenses.

In the scientific literature, there is a broad consensus regarding the important role of vitamin E and Se alone in human health and diseases [248,249]. Other papers also highlight the potential protective effect of both compounds in skin aging [136,187]. As lipids are involved in the epidermal barrier and regulate its permeability, physical properties and antimicrobial defense, oxidative damages due to intrinsic and extrinsic sources will contribute to skin aging and disorders [250]. From a mechanistic point of view, vitamin E sustains Se and vice versa since they contribute to the elimination of lipid peroxides resulting from the interaction of excessive ROS with polyunsaturated fatty acids (Figure 5). The first one acts as a direct scavenger of the lipid radical, the second one as a co-factor of GPx enzyme destroying lipid peroxides. Interestingly, many animal studies more particularly in veterinary medicine have reported a beneficial effect with the Se/vitamin E combination [251,252,253,254,255,256]. In mice, Burke et al. [140] evidenced that a topical L-selenomethionine with topical or oral vitamin E significantly reduced acute and chronic UV-induced skin damage. In diabetic rats, a combination of vitamin C (250 mg/kg), vitamin E (250 mg/kg) and Se (0.2 mg/kg) taken by gavage for 30 days reduced skin lipid peroxidation and glycation [256]. However, this corresponds to very high supra nutritional doses in human (2 g vitamin E and 1.8 mg Se). In humans, the association of both selenium/vitamin E taken orally has been poorly investigated. A major study on a large scale was the Selenium and Vitamin E Cancer Prevention Trial (SELECT) who showed that a daily intake of selenium (200 µg)/vitamin E (400 mg) did not prevent prostate cancer [257].

Besides the strong synergistic antioxidant activity, vitamin E and Se also have common properties of glycation, telomere attrition and epigenetic regulation in the skin. Despite these properties, the protective effect of Se/vitamin E combination has been poorly investigated in human studies, as shown in Table S2. Fairris et al. [166] failed to observe clinical improvement in psoriasis patients supplemented daily with 600 µg selenium-enriched yeast and 270 mg (600 IU) vitamin E for 12 weeks. In contrast, oral intake in Se and vitamin E at nutritional doses, but associated with other antioxidants, has been reported to reduce skin damage induced by UV exposure [210,211,216] or to improve the skin quality [202,213,215].

## 9. Discussion

Increased OS from intrinsic or extrinsic sources is well recognized to play a key role in the development of skin disorders and aging. To minimize these deleterious effects, recent papers highlight the link between nutrition and healthy skin. Thus, there is increased evidence that a balanced diet rich in fruits and vegetables containing antioxidants (vitamins C and E, carotenoids, polyphenols) is of great importance for maintaining good skin health [258]. In Europe, the average consumption of fruits and vegetables is 386 g per day (France 342 g) (https://www.eufic.org accessed on 20 September 2020), while the WHO recommendation is to consume ≥400 g of the fruits and vegetables corresponding to five portions per day [259]. However, a recent report showed that 33% of the European population (17.4% in Belgium, 25.2% in France) have a poor consumption (less than 1 portion) of fruits and vegetables [260].

Clearly, we found in the literature different studies showing that low plasma levels in vitamin E and selenium can trigger skin and hair disorders [161,163,176,177]. In this context, it is suggested that oral antioxidants can be good adjuvants to obtain healthier skin and more particularly to protect this organ from short- and long-term UV-induced oxidative damage [261,262,263]. Of all the antioxidants, vitamin E is certainly the one that has been most studied for its skin protective effects. The direct topical application of vitamin E seems to be the classical and safety route for skin protection via its capacity to eliminate lipid peroxides [185,191,264]. Oral use of vitamin E exhibits photo protection at supra nutritional doses (Table S2). When associated with vitamin C at high doses, its effect was amplified [195,196,208,209].

In the present paper, we showed that both vitamin E and Se exhibit, from a mechanistic point of view, a wide range of common effects beyond their antioxidant action to protect skin against oxidative stress (Figure 5). In most human studies reported in Table S1, oral vitamin E intake is given daily at doses (180–2000 mg) that are largely higher than the NVR (12 mg) commonly used in the majority of supplements. Interestingly, Oh et al. [203] reported that vitamin E intake from the diet may reduce the risk of developing atopic dermatitis. In the case of high doses in vitamin E, this therefore requires some caution since it must be kept in mind that ROS at the physiological level exerts important physiological effects in cells [24,25,26]. Taking vitamin E at a high dose, even if considered safe, could induce, over a long period, an adverse effect by scavenging physiological ROS in cells, leading therefore to inhibition of the protective mechanisms for the body. Long term supplementation at supra-nutritional doses (>180 mg or 400 IU) leads to increased mortality [265]. Same observations have been made with Se at 300 µg/d (two over five years [266]. More specifically, Se supplementation at a high dose is not recommended in diabetic patients, since it has been shown that such pathology by itself induces a high plasma Se concentration [125]. In a general way, to take antioxidant supplements at a supra nutritional dose, it is therefore strongly recommended to do so on medical advice with the support of a blood test to check the basal antioxidant level.

Notably, oral intake of the combination vitamin E/Se at nutritional doses can exhibit photoprotective effects, but only when combined with other antioxidants, such as carotenoids [211,213] and polyphenols [214,215]. The polyphenols family includes phenolic acids and flavonoids (anthocyanins, flavanones, flavonols, and flavones). Their role in skin health, aging and photoprotection has been recently reviewed in an extensive way [15,267,268,269,270]. Among their mechanistic effects, polyphenols have, in contrast to other antioxidants, this unique capacity to stimulate the Keap1/Nrf2/ARE pathway [31,271,272], and more particularly, in the skin leading to the expression of genes coding for the important antioxidant enzymes [273,274] (Figure 1). These enzymes react a thousand to a million times more rapidly with ROS than small antioxidant molecules do. This results in a significant resistance at cellular levels to a subsequent highly lethal dose of oxidants. Recently, Ogawa and Ishitsuka [275] reviewed the role of Keap1-Nrf2 system in the pathophysiology of atopic dermatitis and psoriasis and the therapeutic approaches that utilize this system.

In a recent review, Bocheva et al. [276] reported that active metabolites of vitamin D3 can protect skin against pollution, UVB and microbial infections, notably through their antioxidant properties, even if controversial [277]. Consequently, as subjects suffering from photosensitive disorders must avoid sun exposure, they are at risk of vitamin D deficiency. Maintaining a vitamin D serum concentration within normal levels using supplements could therefore be of interest in atopic dermatitis, psoriasis, vitiligo, polymorphous light eruption, mycosis fungoides, alopecia areata, systemic lupus erythematosus, and melanoma patients [278,279]. To the best of our knowledge, no study to date has examined the effects of the combination of vitamin E/Se in skin aging and disorders.

## 10. Conclusions

The combination of vitamin E/Se taken by an an oral route represents an important piece in the puzzle of antioxidants allowing for the protection of skin against diseases and aging. They act in synergy to eliminate the lipid peroxides involved in skin disorders. Moreover, both molecules also down regulate other important mechanisms, such glucooxidation, metalloproteinase expression, telomere attrition and DNA methylation. However, a main question is to determine at which doses they could exert, in vivo, skin protection, namely nutritional or supra-nutritional ones. In the photo-aging process, Granger et al. [215] suggest that oral supplementation at nutritional doses could potentially be an adjuvant for sunscreens. Clinical trials on a larger scale are needed to explore in detail the real beneficial effect of this combination alone or associated with other antioxidant compounds. It is important to always keep in mind that the intake of high doses of antioxidants must be done under strict medical control.

Oxidative stress is thought to be involved in hair aging. However, there is little data about a potential protective effect by oral supplementation of vitamin E and Se. Almohanna et al. [228] concluded, in a recent broad literature search, that large double-blind placebo-controlled trials are required to determine the potential effect of supplementation of specific micronutrients, such as vitamin E and selenium on hair growth in people with both micronutrient deficiency and non-scarring alopecia in order to establish any association between hair loss and such micronutrient deficiency. Studies demonstrating hair loss as middle- and long-term sequela in COVID-19 patients characterized by depletion in plasma antioxidants and trace elements could provide some information about the potential beneficial effect of oral antioxidant supplementation.

## Supplementary Materials

The following supporting information can be downloaded at: https://www.mdpi.com/article/10.3390/antiox11112270/s1, Table S1: Overview of stfudies related to oral intake in vitamin E in skin aging and disorders. Table S2: Overview of studies related to oral intake in vitamin E combined with Se and other antioxidants in skin aging and disorders.

## Abbreviations

AA	alopecia aerata
AE	antioxidant efficacy
AGA	androgenetic alopecia
AGEs	advanced glycation end products
AMK	N1-acetyl-5-methoxykynuramine
AFMK	N1-acetyl-N2-formyl-5-methoxykynuramine
AP-1	activator protein
ARE	Antioxidant Response Element
CV	cyclic voltametry
Dis	3-deiodinases
DNA	deoxyribonucleic acid
DNAm	deoxyribonucleic acid methylation
DNDMT	DNA demethyltransferase
DNMT	DNA methyltransferase
GPx	glutathione peroxidase
GSH	glutathione
GSSG	oxidized glutathione
HAT	histone acetyltransferase
HDAC	Histone deacetylase
HDMT	histone demethyltransferase
HMT	histone methyltransferase
H_2_O_2_	hydrogen peroxide
hTERT	telomerase reverse transcriptase
Keap 1	Kelch-like ECH-associated protein 1
MDA	malonaldehyde
MED	minimal erythrema dose
MFRTA	Mitochondrial Free Radical Theory of Aging
MMP	matrix metalloproteinase
NF-κB	nuclear factor kappa B
Nrf2	Nuclear factor (erythroid-derived 2)-like 2
O_2_	oxygen
8-OHdG	8-hydroxy-2′-deoxyguanosine
8-oxoGua	8-oxo-7,8-dihydroguanine
OS	oxidative stress
OSWV	osteryoung square wave voltammetry
PI3-K	phosphatidylinositol 3-kinase
PKC	protein kinace C
R^•^	lipidic radical
RH	polyunsaturated free fatty acid
ROO^•^	peroxyl radical
ROOH	lipid peroxide
ROS	reactive oxygen species
SC	striatum corneum
-SH	thiol group
SIRT1-7	silencing information regulator 1-7
SM	sodium selenomethionine
Se	selenium
SeCyS	selenocysteine
SELECT	Selenium and Vitamin E Cancer Prevention Trial
SePP1 or SELENOP	selenoprotein P
SOD	superoxide dismutase
SS	sodium selenite
TE	telogen effluvium
TRxR1, TRxR2, TRxR3	Thioredoxin reductases 1, 2, 3
UVA and UVB	ultraviolet A and B
Vit C	vitamin C
Vit C^•^	ascorbyl radical
Vit E	vitamin E
Vit E^•^	tocopheryl radical
WHO	World Health Organization
